# Biophysical and transcriptomic characterization of LL-37-derived antimicrobial peptide targeting multidrug-resistant *Escherichia coli* and ESKAPE pathogens

**DOI:** 10.1038/s41598-025-22890-7

**Published:** 2025-10-16

**Authors:** Omar Eladl

**Affiliations:** Faculty of Pharmacy, King Salman International University, Ras Sudr, Egypt

**Keywords:** Antimicrobial peptide, Multidrug resistance, Membrane disruption, Cytotoxicity, Transcriptomics, Biochemistry, Biological techniques, Biotechnology, Drug discovery, Microbiology

## Abstract

The emergence of multidrug-resistant (MDR) bacterial pathogens is an alarming global health threat that demands new therapeutic strategies beyond conventional antibiotics. Here, we present a rationally designed antimicrobial peptide (AMP) derived from mammalian cathelicidins and defensins that selectively targets bacterial membranes with low cytotoxicity toward mammalian cells. Circular dichroism spectroscopy revealed that the peptide adopts an α-helical conformation upon membrane interaction, a key feature of its mechanism. Surface plasmon resonance and isothermal titration calorimetry demonstrated high-affinity and selective binding to bacterial lipid membranes. Functionally, the peptide was strongly bactericidal against clinical MDR *Escherichia coli* (*E. coli*) and clinically important ESKAPE pathogens (*Enterococcus faecium, Staphylococcus aureus, Klebsiella pneumoniae, Acinetobacter baumannii, Pseudomonas aeruginosa,* and *Enterobacter* spp.). Compared with the parent peptide LL-37, our AMP exhibited lower minimum inhibitory concentrations (MICs) and faster bactericidal kinetics across both Gram-negative and Gram-positive strains. Calcein leakage assays, showing effective membrane disruption. Importantly, cytotoxicity experiments with human epithelial (Caco-2) and immune (THP-1) cells indicated low cytotoxicity at concentrations exceeding bactericidal levels, supporting a favorable therapeutic window. ELISA quantifications of cytokines (IL-6, TNF-α) further suggested immunomodulatory effects at bactericidal concentrations. Transcriptomic profiling of *E. coli* treated with sub-lethal concentrations of the peptide exhibited upregulation of bacterial stress response pathways and downregulation of vital metabolic processes, reflecting the complex antimicrobial action of the peptide. Collectively, these findings highlight this LL-37-derived AMP as a promising candidate for treating MDR bacterial infections caused by *E. coli* and ESKAPE pathogens and for guiding the development of next-generation antimicrobial agents.

**Supplementary Information:**

The online version contains supplementary material available at 10.1038/s41598-025-22890-7.

## Introduction

The global spread of multidrug-resistant (MDR) bacterial infections poses a significant and escalating threat to public health infrastructures worldwide^1^. Widespread and, in certain instances, uncontrolled use of antibiotics in both clinical medicine and agriculture has spurred the growth of antibiotic-resistant bacteria, weakening existing antimicrobial therapies^2–4^. This issue is particularly concerning for *E. coli*, a ubiquitous Gram-negative bacterium that is both a normal gut commensal and an opportunistic pathogen^5^. MDR *E. coli* isolates cause serious infections, such as urinary tract infections and neonatal meningitis, leading to life-threatening complications, including bacteremia and sepsis, prolonged hospital stays, and increased morbidity, mortality, and economic burden^6,7^. The ability of *E. coli* and other bacteria to acquire and disseminate resistance-promoting genes, including extended-spectrum beta-lactamases and carbapenemases, creates further obstacles to therapy, pointing to a requirement for new, effective therapeutic agents^8,9^.

Amidst this need, antimicrobial peptides (AMPs) have emerged as an attractive class of drugs due to their broad-spectrum activity, rapid bactericidal activity, and reduced ability to generate resistance^10^. AMPs are evolutionarily conserved components of innate immunity that target microbial membranes via electrostatic attraction, leading to permeabilization and cell death^11,12^. In addition, AMPs exhibit immunomodulatory activities, including the regulation of inflammatory responses and promotion of wound healing^13,14^.

However, despite their potential, several limitations hinder the clinical application of natural AMPs^15^, including poor selectivity leading to host toxicity, susceptibility to protease degradation, low stability under physiological conditions, and challenges in large-scale synthesis^16,17^. Translating AMPs into clinically approved therapeutics remains difficult due to rapid proteolytic degradation, poor bioavailability, potential host toxicity, and high manufacturing costs^10,18^. Nevertheless, several AMPs^19^, such as LL-37, pexiganan, and omiganan, have progressed into clinical trials, demonstrating safety and efficacy in treating skin infections and chronic wounds. These experiences guide the design of next-generation peptides that balance antimicrobial potency with pharmacokinetic stability and tolerability, underscoring the translational potential of rationally engineered AMPs^16^.

To overcome these barriers, rational design strategies for artificial AMPs have gained popularity, making use of advances in peptide chemistry, structural biology, and computational modeling^20,21^. By modulating a few physicochemical parameters, i.e., net positive charge, hydrophobicity, amphipathicity, and secondary structure, artificial AMPs can be engineered for increased selectivity, potency, and stability^22^. In particular, α-helical amphipathic peptides based on human host defense peptides such as cathelicidins (LL-37) and defensins have exhibited appreciable therapeutic potential^23,24^. These peptides typically adopt random coil conformations in solution but become α-helices upon binding negatively charged bacterial membranes, enabling membrane insertion and disruption^25,26^. Achieving optimal antimicrobial activity requires balancing hydrophobicity and positive charge to maximize bacterial targeting with minimal host toxicity.

**Fig. 1:**
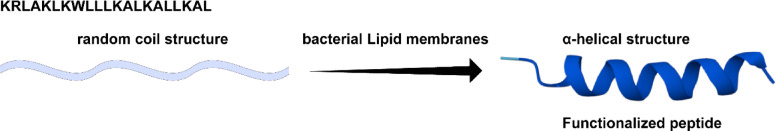
Schematic illustration of the peptide’s conformational transition from a random coil in solution to an α-helical structure upon binding to the bacterial lipid membrane.

We herein rationally designed and synthesized a novel artificial AMP with amphipathic α-helical motifs inspired by human cathelicidins and defensins to achieve maximal targeting of bacteria with minimal host toxicity (Fig. 1). The peptide sequence (KRLAKLKWLLLKALKALLKAL) was engineered by mimicking the α-helical amphipathic structure of LL-37, incorporating a strategic arrangement of cationic residues (lysine and arginine) and hydrophobic residues (leucine, alanine, tryptophan) to create a polar–nonpolar distribution across the helix. The use of helical wheel projections and secondary structure predictions ensured that this arrangement promotes membrane insertion similar to LL-37. We conducted an extensive characterization of the peptide’s structural and functional behaviors to elucidate how its biophysical properties are related to antimicrobial activity. Circular Dichroism (CD) spectroscopy was used to investigate the secondary structure of the peptide in aqueous solution and membrane-mimetic conditions and to show inducible α-helical conformations that are required for membrane interaction. Surface Plasmon Resonance (SPR) and Isothermal Titration Calorimetry (ITC) provided quantitative measures of the binding affinity and specificity of the peptide for bacterial membrane lipids compared with mammalian-like membranes, defining selective targeting mechanisms. Functional microbiological assays quantified bactericidal activity against MDR *E. coli* clinical isolates that display tight, rapid killing and low resistance induction after repeated exposures. Importantly, the peptide also showed activity against other clinically relevant Gram-positive and Gram-negative pathogens, including ESKAPE species, and exhibited improved potency compared with LL-37. Besides, cytotoxicity and immunomodulatory activity were investigated on human epithelial (Caco-2) and immune (THP-1) cells, showing low cytotoxicity and positive modulation of pro-inflammatory cytokines, indicating good safety.

For greater mechanistic understanding, transcriptomic analysis was performed on sub-inhibitory concentration-treated *E. coli*. This revealed extreme alterations to bacterial gene expression, including induction of stress response mechanisms and repression of key metabolic processes, shedding light on the intracellular impacts triggered by membrane disruption and peptide internalization.

Together, these aggregate biophysical, microbiological, and molecular data highlight the therapeutic value of this artificial AMP as a novel antimicrobial drug. Our findings present valuable information for the rational design of next-generation AMPs capable of addressing the pressing clinical demand for MDR bacterial infections, ultimately resulting in safer and more effective antimicrobial therapeutics.

## Materials and methods

### Peptide design and synthesis

The AMP, KRLAKLKWLLLKALKALLKAL, was rationally designed using the conserved amphipathic α-helical domain (residues 17–29, sequence: FKRIVQRIKDFLR) of human LL-37 (UniProt ID: P49913) as the starting scaffold. This region was chosen for its strong α-helical propensity, membrane activity, and amphipathic nature, making it a critical functional core in many cathelicidin-derived antimicrobial peptides.

To enhance antimicrobial potency and reduce cytotoxicity to mammalian cells, we implemented a structure-guided, stepwise optimization strategy focused on three key parameters: net positive charge, hydrophobic moment, and helical amphipathicity (Supplementary Fig. 1).

First, positively charged residues (lysine and arginine) were strategically introduced and conserved to boost electrostatic attraction to negatively charged bacterial membranes, resulting in a net charge of + 7. Second, hydrophobic residues were optimized to achieve a hydrophobic moment of 0.532, supporting efficient membrane interaction without compromising selectivity. The HeliQuest web server (http://heliquest.ipmc.cnrs.fr) was used to analyze peptide physicochemical properties, confirming ideal helical projection with distinct hydrophilic and hydrophobic faces. Tertiary structure predictions were generated using I-TASSER (https://zhanggroup.org/I-TASSER/), which predicted a stable α-helix conformation with a C-score of –0.79, indicating reliable folding into a helix suitable for membrane insertion. Antimicrobial potential was assessed using the CAMPR3 predictor (http://www.camp.bicnirrh.res.in/), which classified the peptide as highly antimicrobial with a probability score of 0.92 using the SVM model. Through this iterative, rational design approach, KRLAKLKWLLLKALKALLKAL was selected as the lead AMP candidate due to its optimized amphipathic structure, strong predicted membrane disruption capacity, and low predicted mammalian cytotoxicity.

Solid-phase peptide synthesis (SPPS) was performed under Fmoc (9-fluorenylmethoxycarbonyl) chemistry on a Liberty Blue automated synthesizer (CEM Corporation). Standard coupling cycles utilized HBTU/HOBt activation with DIPEA as base. Final assembly of the peptide chain was cleaved from the resin and side chains deprotected with a trifluoroacetic acid (TFA)-based cleavage cocktail that consisted of 95% TFA, 2.5% triisopropylsilane, and 2.5% water for 3 h at room temperature.

The crude peptide was precipitated upon the addition of cold diethyl ether, recovered by centrifugation at 4000 rpm for 10 min at 4 °C, and subsequently lyophilized. The purification was achieved using preparative reverse-phase high-performance liquid chromatography (RP-HPLC) on a C18 column (Phenomenex Jupiter, 250 × 10 mm, 10 μm particle size) with a linear gradient of acetonitrile in water with 0.1% TFA from 5 to 60% over 60 min at a flow rate of 4 mL/min. The purified peptide was determined to be > 95% pure by analytical HPLC. Molecular weight and identity were verified by matrix-assisted laser desorption/ionization time-of-flight (MALDI-TOF) mass spectrometry (Bruker Autoflex) using α-cyano-4-hydroxycinnamic acid (CHCA) as the matrix, confirming the predicted mass of 2,470 Da.

### Circular dichroism (CD) and synchrotron radiation CD (SRCD)

CD spectra were recorded on a Jasco J-1500 spectropolarimeter at 25°C using a 0.1 cm quartz cuvette. Peptide stock solutions were prepared at 50 µM in 10 mM sodium phosphate buffer (pH 7.4). To simulate membrane conditions, 30% (v/v) trifluoroethanol (TFE), a helix-stabilizing cosolvent, was added. Large unilamellar vesicles (LUVs) composed of POPE/POPG (7:3 molar ratio) or POPC/cholesterol (7:3 molar ratio) were used to mimic bacterial and mammalian membranes, respectively. POPE (1-palmitoyl-2-oleoyl-sn-glycero-3-phosphoethanolamine) and POPG (1-palmitoyl-2-oleoyl-sn-glycero-3-phospho-(1'-rac-glycerol)) represent major constituents of Gram-negative bacterial membranes, while POPC (1-palmitoyl-2-oleoyl-sn-glycero-3-phosphocholine) and cholesterol are key components of mammalian plasma membranes. LUVs were prepared by extrusion through 100 nm polycarbonate membranes at a total lipid concentration of 10 mM, with peptide-to-lipid molar ratios of 1:50.

CD spectra were collected from 190–260 nm, with a 1 nm bandwidth and averaged over three scans. For thermal denaturation, spectra were recorded at 5°C intervals from 10°C to 90°C, followed by cooling to assess refolding reversibility.

Secondary structure analysis was conducted using the DichroWeb server with the CONTIN algorithm and reference set to estimate α-helicity and other structural elements from CD spectra. This allowed objective comparison of structural transitions under different environmental conditions. SRCD spectra were recorded on the B23 beamline at Diamond Light Source. Samples (100 µM peptide) were set up in identical buffer and lipid conditions, and spectra were collected to give bigger signal-to-noise ratios and extend below 190 nm for improved resolution of secondary structure^27^.

### Surface plasmon resonance (SPR)

Lipid-coated sensor chips were prepared on an L1 chip (Cytiva) on a Biacore T200 instrument. Small unilamellar vesicles (SUVs) composed of POPE/POPG (7:3) or POPC/cholesterol (7:3) were immobilized on the chip surface through vesicle fusion at a lipid concentration of 0.5 mg/mL in the HBS-EP buffer (10 mM HEPES, 150 mM NaCl, 3 mM EDTA, 0.005% surfactant P20, pH 7.4). The peptide was analyzed at concentrations ranging from 0.1 to 10 µM in a running buffer at 25°C. Association and dissociation phases were monitored for 180 and 300 s, respectively. Sensorgrams were referenced in duplicate against blank channels and buffer injections. Kinetic parameters (association rate constant k_a_, dissociation rate constant k_d_) and equilibrium dissociation constants (K_D_) were calculated by fitting data to a Langmuir 1:1 binding model with Biacore T200 Evaluation software version 3.1^28,29^.

### Isothermal titration calorimetry (ITC)

ITC experiments were conducted on a MicroCal PEAQ-ITC instrument (Malvern Panalytical) at 25°C. Large unilamellar vesicles of POPE/POPG (7:3) were prepared at 0.5 mM total lipid in 10 mM phosphate buffer (pH 7.4). The peptide stock solution was prepared at 100 µM in the same buffer. Titration of the peptide solution into the lipid suspension was done in 19 injections of 2 µL each with 150-s spacing and 4-s injection time for equilibration. The heat of dilution was accounted for by control titration of the peptide into the buffer. Data were analyzed with MicroCal PEAQ-ITC analysis software, with the data being fitted to a single-site binding model to assess binding enthalpy (ΔH), entropy changes (ΔS), binding constant (Kb), and stoichiometry (n)^30^.

### Antimicrobial assays

Minimum inhibitory concentrations (MICs) and minimum bactericidal concentrations (MBCs) were determined according to the Clinical and Laboratory Standards Institute (CLSI) guidelines. Bacterial strains used in this study included *E. coli* K-12 (ATCC® 10798™, ATCC), uropathogenic *E. coli* CFT073 (ATCC® 700928™, ATCC), multidrug-resistant (MDR) *E. coli* NCTC 13353™ (NCTC), *Pseudomonas aeruginosa* (ATCC® 27853™, ATCC), *Acinetobacter baumannii* (ATCC® 19606™, ATCC), *Klebsiella quasipneumoniae* (ATCC® 700603™, ATCC), *Staphylococcus aureus* (ATCC® 25923™, ATCC), and *Enterococcus faecium* (ATCC® 19434™, ATCC). Bacteria were grown in Mueller–Hinton broth (MHB) to mid-log phase (OD600 ≈ 0.5), then diluted to 5 × 10^5^ CFU/mL for MIC and MBC testing.

Peptides, including the designed peptide and LL-37, were prepared in sterile water and serially diluted twofold from 100 µM to 0.1 µM in 96-well microtiter plates. For MIC determination, 100 µL of bacterial suspension was added to each well containing 100 µL of peptide dilution, and plates were incubated at 37°C for 18 h. MIC was defined as the lowest peptide concentration at which no visible bacterial growth was observed.

For MBC determination, 10 µL aliquots from wells at and above the MIC were plated onto Mueller–Hinton agar and incubated at 37°C for 24 h. MBC was defined as the lowest concentration killing ≥ 99.9% of bacteria.

Time-kill assays were conducted to assess bactericidal kinetics. Bacteria were incubated with peptides at four times the MIC, and samples were collected at 0, 0.5, 1, 2, 4, and 6 h. Collected samples were serially diluted in phosphate-buffered saline (PBS) and plated on Mueller–Hinton agar for CFU enumeration after 24 h of incubation at 37°C. Killing kinetics were evaluated for *E. coli* K-12, uropathogenic *E. coli*, MDR *E. coli*, as well as additional Gram-negative species (*P. aeruginosa*, *A. baumannii*, *K. quasipneumoniae*) and Gram-positive species (*S. aureus*, *E. faecium*). Both LL-37 and the designed peptide were tested in parallel under identical conditions to allow direct comparison of antimicrobial potency and bactericidal speed.

### Cytotoxicity and Immunomodulation

Human intestinal epithelial Caco-2 cells and monocytic THP-1 cells were cultured in Dulbecco’s Modified Eagle Medium (DMEM) and Roswell Park Memorial Institute medium (RPMI 1640), respectively, supplemented with 10% fetal bovine serum and 1% penicillin–streptomycin. Cells were plated in 96-well plates at 1 × 10^4^ cells/well (Caco-2) and 5 × 10^4^ cells/well (THP-1). After overnight incubation, cells were incubated with peptide concentrations of 5 to 50 µM for 6 h at 37°C and 5% CO_2_. Cell viability was assessed with the MTT assay by measuring absorbance at 570 nm and lactate dehydrogenase (LDH) release membrane integrity assays^31^. Cell viability values were normalized to untreated controls, while LDH release values were normalized to 0.1% Triton X-100, which represented 100% release.

For immunomodulation assays, THP-1 cells were pre-stimulated with lipopolysaccharide (LPS, 100 ng/mL) for 4 h to induce proinflammatory cytokine production before peptide treatment. The concentrations of interleukin-6 (IL-6) and tumor necrosis factor-alpha (TNF-α) cytokines in supernatants of THP-1 cultures were assessed by enzyme-linked immunosorbent assay (ELISA) kits (R&D Systems) according to the manufacturer’s protocol^32^. Cytokine concentrations were normalized to LPS-stimulated controls to determine relative modulation by peptide treatment.

### Membrane disruption mechanism

Leakage assays using large unilamellar vesicles (LUVs) with 70 mM calcein dye (self-quenching concentration) in 10 mM phosphate buffer, pH 7.4, were performed. The free dye was removed by gel filtration on Sephadex G-50 columns. Vesicles were incubated at 25°C in the presence of peptide concentrations of 10 µM. Fluorescence was measured at 490/520 nm excitation/emission using a plate reader (Tecan Infinite M200). Percent leakage was calculated relative to complete lysis by 0.1% Triton X-100 detergent.

### Transcriptomic analysis

For transcriptomic profiling, *E. coli* MDR strain cultures were grown to the mid-log phase (OD_600_ ≈ 0.5) and treated with sub-inhibitory concentrations of peptide (0.5 × MIC) for 1 h at 37°C, shaking, to capture early stress responses without inducing extensive cell death or secondary effects. Untreated cultures served as the controls. Total RNA was purified using the RNeasy Mini Kit (Qiagen) with on-column digestion by DNase. The integrity of the RNA was established by Agilent 2100 Bioanalyzer, with the RNA Integrity Numbers (RIN) > 8.0. Libraries were made using the NEBNext Ultra II Directional RNA Library Prep Kit for Illumina with rRNA depletion. Sequencing was performed on an Illumina NovaSeq 6000 platform to generate paired-end 150 bp reads, with an average depth of 20 million reads per sample across three biological replicates. FastQC and Trimmomatic were used for quality control and trimming. Reads were mapped to the *E. coli* reference genome (NCBI accession NC_000913.3) using HISAT2^33^. Differential gene expression analysis was performed with DESeq2 employing a false discovery rate (FDR) cutoff of 0.05 and ± 1.5 log2 fold change threshold. Statistically controlled genes were assigned and normalized to the Kyoto Encyclopedia of Genes and Genomes (KEGG) pathway database with the ClusterProfiler R package, to identify disrupted biological pathways and provide quantitative enrichment insights into the functional impact of peptide treatment, with enrichment significance calculated using a hypergeometric test and adjusted for multiple comparisons using the Benjamini–Hochberg FDR method^34^.

### Data analysis

Experiments were conducted in three independent biological replicates performed. Triplicate biological replicates were selected to ensure reproducibility while maintaining experimental feasibility.

## Results

### Peptide structure and stability

**Fig. 2:**
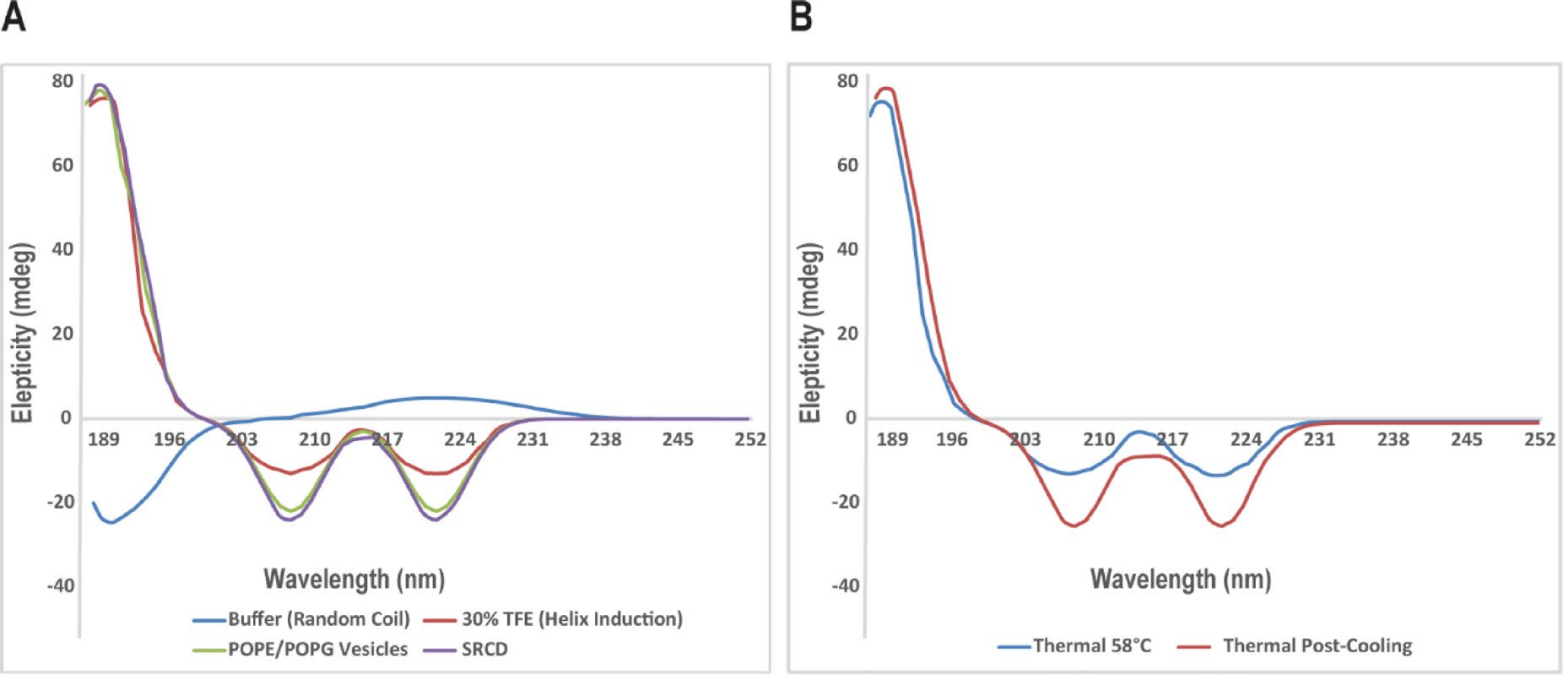
Secondary structure analysis of the peptide by CD and SRCD spectroscopy. **(A**) CD spectra show a random coil conformation in phosphate buffer (10 mM, pH 7.4), induction of α-helicity in 30% TFE, and enhanced helical content upon interaction with POPE/POPG (7:3) LUVs, mimicking bacterial membranes. SRCD confirmed these structural transitions with higher spectral resolution. Spectra represent mean ellipticity (mdeg) ± SD from three independent experiments. Statistical analysis by one-way ANOVA confirmed significant differences in α-helicity compared to buffer (p < 0.001). **(B)** Thermal denaturation monitored by CD reveals a cooperative unfolding transition around 58°C, with full recovery upon cooling, indicating reversible α-helix folding. Data are mean ± SD of three replicates.

To determine the structural features of the investigated peptide, we first performed CD spectroscopy under various conditions that mimic physiological and membrane environments. In aqueous phosphate buffer (10 mM, pH 7.4), the CD spectra indicated that the peptide was predominantly present as a random coil structure, characterized by a minimal negative ellipticity at 222 nm (Fig. 2A). However, upon the addition of 30% trifluoroethanol (TFE), a stabilizer of α-helix structure solvent known to induce stabilization of α-helical structure, we observed significant induction of α-helicity as reflected by the appearance of characteristic double minima at 208 nm and 222 nm. The shift confirmed the peptide’s intrinsic capacity to adopt an amphipathic α-helix conformation in a membrane-mimetic environment (Fig. 2A).

To improve the modeling of bacterial membrane interactions, the peptide was incubated with large unilamellar vesicles (LUVs) composed of POPE (1-palmitoyl-2-oleoyl-sn-glycero-3-phosphoethanolamine) and POPG (1-palmitoyl-2-oleoyl-sn-glycero-3-phosphoglycerol) in a 7:3 molar ratio. These phospholipids are commonly used to simulate the anionic outer membranes of Gram-negative bacteria. CD spectra in this setting showed even more distinct α-helical signatures, consistent with stable membrane-induced folding (Fig. 2A). Deconvolution of CD spectra using DichroWeb revealed an estimated α-helical content of 69% in POPE/POPG vesicles, compared to 18% in aqueous buffer, and 57% in 30% TFE, indicating that the peptide adopts a highly ordered conformation specifically in bacterial-like lipid environments.

SRCD spectroscopy was subsequently implemented at Diamond Light Source with the objective of enhanced spectral resolution and sensitivity. SRCD spectra corroborated the traditional CD results but provided more definitive evidence for the peptide’s greater helical stability in the bacterial-like lipid environment, confirming the peptide’s selective interaction with membranes (Fig. 2A).

Thermal denaturation experiments, where the temperature was raised stepwise from 10°C to 90°C, showed a cooperative unfolding transition around 58°C, as expected for a moderately stable helical structure. Interestingly, reversal of cooling the sample back to 10°C reproduced the initial spectrum, demonstrating reversible folding behavior (Fig. 2B).

### Biophysical interactions with model membranes

**Fig. 3:**
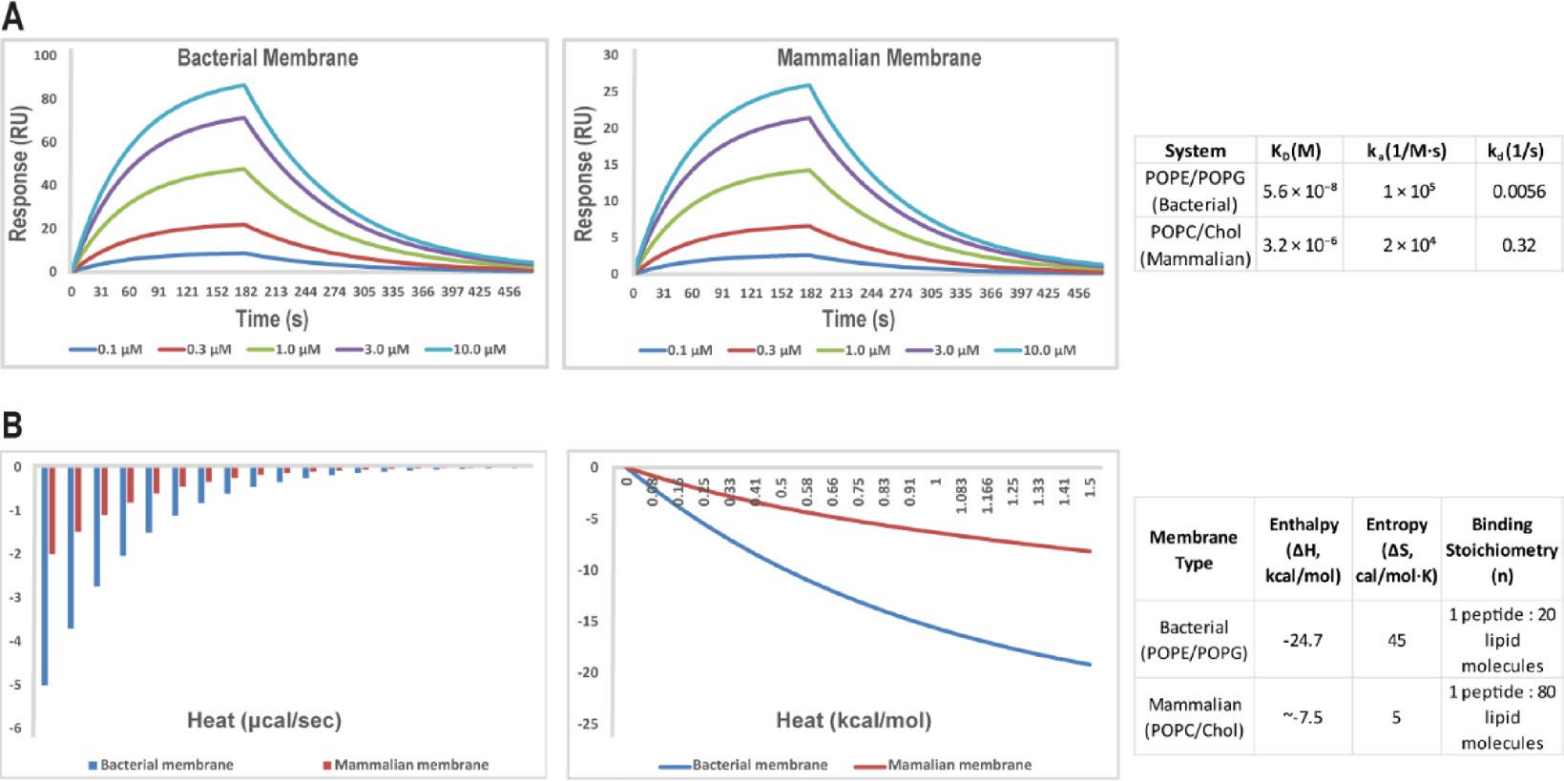
Binding selectivity of the peptide for bacterial versus mammalian membrane mimics assessed by SPR and ITC. **(A)** Surface plasmon resonance (SPR) analysis shows distinct binding profiles of the peptide to lipid-coated sensor chips mimicking bacterial (POPE/POPG) and mammalian (POPC/cholesterol) membranes. A table summarizes the kinetic parameters derived from the fits. Sensorgrams represent the mean values of three independent experiments. **(B)** Isothermal titration calorimetry (ITC) measurements reveal strong, thermodynamically favorable binding to bacterial-mimicking vesicles, with minimal interaction observed for mammalian-mimicking vesicles. A summary of thermodynamic parameters and binding stoichiometries is provided in the accompanying table. The thermodynamic parameters shown are mean ± SD from three replicates.

To evaluate the peptide’s binding selectivity and affinity for bacterial versus mammalian membranes, we conducted SPR and ITC assays using lipid-coated sensor chips and vesicles (Fig. 3). The peptide exhibited high-affinity binding to bacterial membrane-mimicking POPE/POPG vesicles with an equilibrium dissociation constant (K_D_) of 5.6 × 10⁻⁸ ± 0.8 × 10⁻⁸ M, an association rate constant (k_a_) of 1.0 × 10^5^ ± 0.2 × 10^5^ M⁻^1^s⁻^1^, and a dissociation rate constant (k_d_) of 5.6 × 10⁻^3^ ± 0.7 × 10⁻^3^ s⁻^1^ (Fig. 3A).

In contrast, binding to mammalian membrane analog POPC/cholesterol vesicles was significantly weaker, with a K_D of 3.2 × 10⁻⁶ ± 0.4 × 10⁻⁶ M, a k_a_ of 2.0 × 10^4^ ± 0.3 × 10^4^ M⁻^1^s⁻^1^, and a k_d_ of 3.2 × 10⁻^1^ ± 0.05 s⁻^1^, reflecting approximately 50-fold lower affinity, slower association kinetics, and much faster dissociation kinetics (Fig. 3A). ITC analysis revealed that binding to POPE/POPG vesicles was strongly exothermic (ΔH = -24.7 ± 1.5 kcal/mol) with a positive entropy change (ΔS =  + 45 ± 3 cal/mol·K), consistent with electrostatic attraction between the cationic peptide and anionic lipids combined with hydrophobic interactions. The binding stoichiometry was approximately one peptide per 20 ± 2 lipid molecules, indicating partial membrane insertion and surface binding (Fig. 3B). By comparison, binding to POPC/cholesterol vesicles displayed a markedly reduced enthalpy change (ΔH ≈ -7.5 ± 0.8 kcal/mol) and negligible entropy gain (ΔS ≈ + 5 ± 1 cal/mol·K), with 1:80 ± 5 binding stoichiometry, indicative of weaker and less cooperative interactions with the mammalian membrane mimic. These data demonstrate the peptide’s strong preference for bacterial membrane lipids, a critical feature for minimizing host toxicity (Fig. 3B).

### Potent antimicrobial activity against bacterial strains

**Fig. 4:**
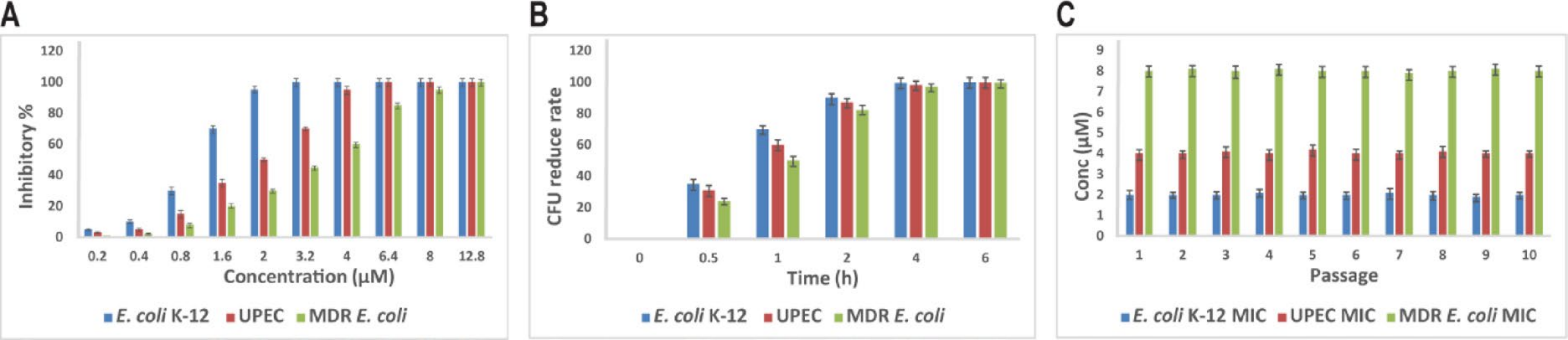
Antimicrobial efficacy of the peptide against various *Escherichia coli* strains. **(A)** Minimum inhibitory concentrations (MICs) determined for laboratory *E. coli* K-12, uropathogenic *E. coli* (UPEC), and multidrug-resistant (MDR) strain, following CLSI guidelines. Data are shown as mean ± SD from three independent biological replicates, with error bars indicating SD. **(B)** Time-kill assays showing bactericidal kinetics at four times the MIC concentration, with 90% bacterial reduction achieved within 2 h for all strains. Values represent mean CFU reduction rate ± SD from three independent replicates; error bars indicate SD. **(C)** Assessment of resistance development after 10 serial passages of each strain in sub-MIC peptide concentrations, indicating no significant increase in MIC and low potential for resistance emergence. MIC values are normalized to passage 1 baseline and presented as mean ± SD from three independent replicates, with error bars indicating SD.

To evaluate the antimicrobial potential of our designed peptide relative to the well-characterized human host defense peptide LL-37, we first focused on a panel of *E. coli* strains, including laboratory *E. coli* K-12, uropathogenic *E. coli* (UPEC), and multidrug-resistant *E. coli* (MDR) isolates. LL-37 is well known for its broad-spectrum antibacterial activity against both Gram-negative and Gram-positive species; therefore, comparing our peptide to LL-37 allows assessment of its enhanced properties as a derivative. Minimum inhibitory concentrations (MICs) demonstrated that the peptide was consistently more potent than LL-37. Specifically, for *E. coli* K-12, the peptide inhibited growth at 2 ± 0.2 µM compared to 6 ± 0.3 µM for LL-37, representing a ~ 67% improvement in potency (p < 0.001). For UPEC, the peptide’s MIC was 4 ± 0.3 µM versus 8 ± 0.5 µM for LL-37, a 50% enhancement, and for MDR *Escherichia coli*, 8 ± 0.5 µM versus 12 ± 0.6 µM, a 33% increase in effectiveness (Fig. 4A and Supplementary Figs. 2A and B).

Time-kill assays further highlighted the peptide’s superior bactericidal kinetics against *E. coli* strains. At four times the MIC, the peptide killed 35 ± 3% of K-12 cells within 30 min, reaching 90 ± 5% at 2 h and near-complete killing (99 ± 2%) at 4 h. In comparison, LL-37 killed only 12 ± 2%, 55 ± 4%, and 70 ± 5% of K-12 cells at the same time points, representing approximately a 2.9-fold faster initial killing rate and ~ 29% higher total killing at 4 h. For UPEC, the peptide killed 28 ± 4% at 30 min, 85 ± 5% at 2 h, and 98 ± 2% at 4 h, whereas LL-37 achieved 10 ± 3%, 50 ± 5%, and 68 ± 4%, respectively, reflecting a markedly faster and more complete clearance. Against MDR, the peptide reduced bacterial counts by 20 ± 3% at 30 min, 78 ± 4% at 2 h, and 96 ± 3% at 4 h, whereas LL-37 achieved only 8 ± 2%, 45 ± 4%, and 65 ± 5% killing at these times. Across all three strains, these differences were statistically significant (p < 0.001) (Fig. 4B and Supplementary Fig. 2C and D), indicating that the peptide achieves faster onset of killing and more efficient bacterial eradication compared to LL-37.

To assess whether this enhanced activity extended beyond *E. coli*, we evaluated additional Gram-negative species (*Pseudomonas aeruginosa*, *Acinetobacter baumannii*, and *Klebsiella pneumoniae*) and Gram-positive pathogens (*Staphylococcus aureus* and *Enterococcus faecium*). Across these strains, LL-37 displayed its characteristic broad-spectrum activity, while the peptide consistently outperformed LL-37 in both MIC and time-kill assays. For example, MICs for the peptide were 6 ± 0.4 µM, 8 ± 0.5 µM, and 4 ± 0.3 µM for *Pseudomonas aeruginosa*, *Acinetobacter baumannii*, and *Klebsiella pneumoniae*, respectively, corresponding to ~ 57%, 50%, and 60% lower MICs than LL-37 (14 ± 0.7, 16 ± 0.8, and 10 ± 0.5 µM; p < 0.001) (Supplementary Fig. 3A). Time-kill assays further confirmed the peptide’s superior bactericidal kinetics: at four times the MIC, it reduced *Pseudomonas aeruginosa* by 40 ± 3% at 30 min, 88 ± 4% at 2 h, and 98 ± 2% at 4 h, compared to LL-37, which achieved only 15 ± 2%, 55 ± 5%, and 72 ± 4% at the same time points (Supplementary Fig. 3B).

Gram-positive species also showed greater susceptibility to the peptide: *Staphylococcus aureus* was inhibited at 2 ± 0.2 µM versus 6 ± 0.3 µM for LL-37 (~ 67% lower MIC), and *Enterococcus faecium* at 4 ± 0.3 µM versus 12 ± 0.6 µM (~ 66% reduction) (Supplementary Fig. 3C). Time-kill experiments mirrored these results, with the peptide inducing ~ 2–threefold faster bacterial killing and higher overall clearance than LL-37 at all measured time points (p < 0.001) (Supplementary Fig. 3D).

Overall, these findings indicate that our peptide, derived from LL-37, exhibits enhanced antimicrobial potency, achieving lower MICs and more rapid bactericidal activity against both *E. coli* strains and a broader panel of Gram-negative and Gram-positive bacteria. Quantitative comparisons demonstrate that structural modifications of LL-37 can improve efficacy by 33–67% in MIC reduction and 2–threefold in bactericidal rate, while maintaining broad-spectrum activity, highlighting the peptide as a promising candidate for further development.

### Cytotoxicity and immunomodulatory effects on human cells

**Fig. 5:**
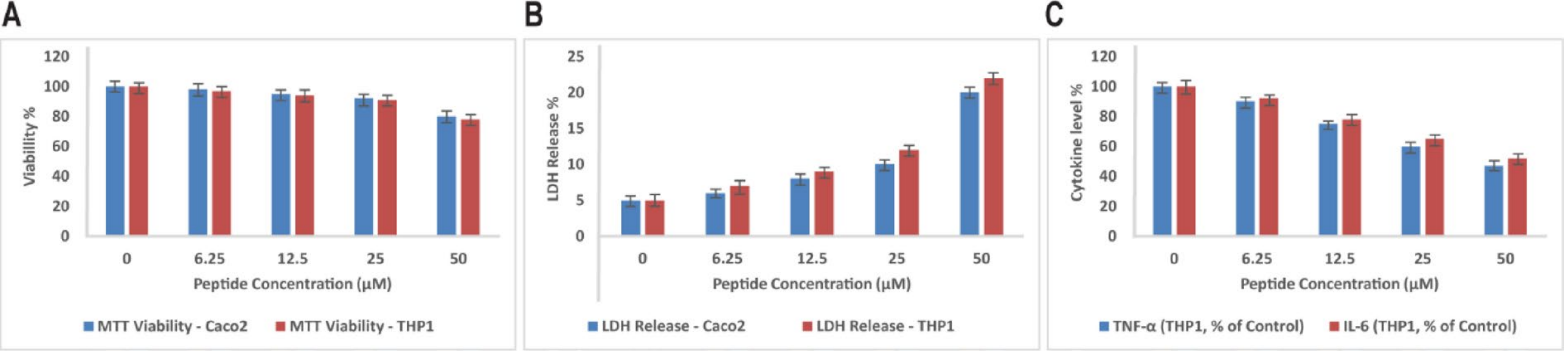
Safety and immunomodulatory assessment of the peptide in human cell models. **(A)** Cell viability of Caco-2 epithelial and THP-1 monocytic cells after 6-h peptide exposure, measured by MTT assay, demonstrating high viability at concentrations up to 25 µM. Values expressed as % relative to untreated controls and shown as mean ± SD from three independent experiments, with error bars indicating SD. **(B)** Lactate dehydrogenase (LDH) release assay indicating minimal membrane disruption at peptide concentrations up to 25 µM, with moderate cytotoxicity observed at 50 µM. Values normalized to 0.1% Triton X-100 (100% release) and presented as mean ± SD from three replicates, with error bars indicating SD. **(C)** Dose-dependent reduction of pro-inflammatory cytokines TNF-α and IL-6 in THP-1 macrophage-like cells, measured by ELISA, suggesting potential anti-inflammatory activity of the peptide. Cytokine levels normalized to LPS-stimulated controls; data shown as mean ± SD from three independent experiments with error bars indicating SD. Statistical significance assessed by one-way ANOVA test (*p* < 0.01).

The peptide’s safety profile was assessed in human epithelial intestinal Caco-2 cells and monocytic THP-1 cells (Fig. 5). Cell viability assays (MTT) showed high viability at concentrations up to 25 µM, corresponding to more than three times the MIC, with values normalized to untreated controls. This supports a favorable therapeutic window (Fig. 5A). At the highest concentration tested, 50 µM, there was moderate cytotoxicity and approximately 20% lactate dehydrogenase (LDH) release, normalized to 0.1% Triton X-100 (100% release), indicating little membrane disruption (Fig. 5B).

Cytokine production was determined by ELISA to monitor immunomodulatory activity. In THP-1 macrophage-like cells, the peptide caused a dose-dependent reduction of pro-inflammatory cytokines, tumor necrosis factor-alpha (TNF-α), and interleukin-6 (IL-6). Cytokine levels were normalized to LPS-stimulated controls, and a concentration-dependent decrease was observed, with reductions of approximately 40% (TNF-α) and 35% (IL-6) at 25 µM. These findings suggest that the peptide may possess anti-inflammatory activity and could help reduce host tissue injury during infection (Fig. 5C).

### Membrane disruption mechanism confirmed by dye leakage

**Fig. 6:**
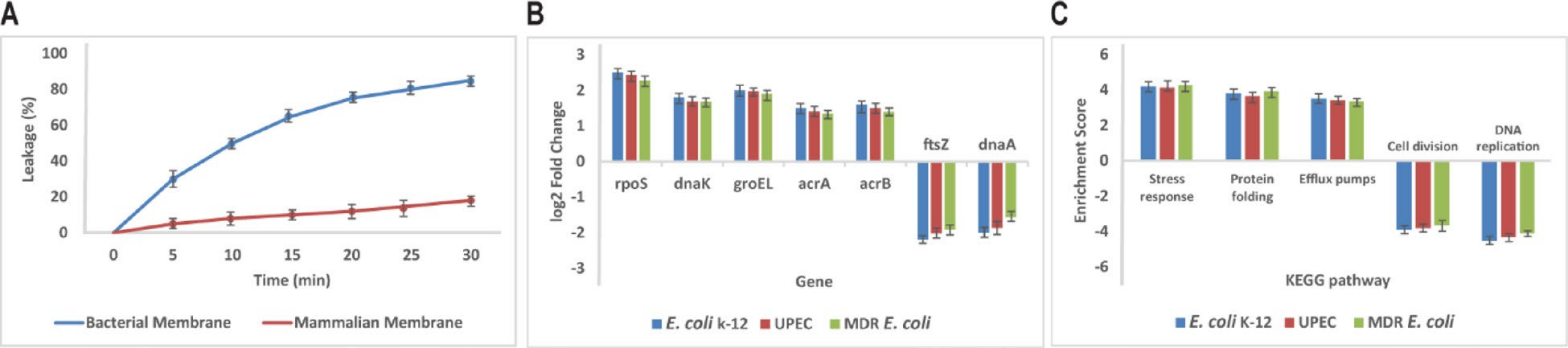
Mechanism of membrane disruption and transcriptomic response to peptide treatment in *E. coli* strains. **(A)** Calcein dye leakage assay demonstrating selective membrane permeabilization of bacterial-mimetic POPE/POPG vesicles by the peptide, with minimal leakage observed in mammalian-mimetic POPC/cholesterol vesicles. Values expressed as % dye leakage relative to 0.1% Triton X-100 (100% release) and shown as mean ± SD from three independent experiments, with error bars indicating SD. **(B)** RNA sequencing analysis showing upregulation of stress response and efflux pump genes and downregulation of growth-related genes in *E. coli* K-12, UPEC, and MDR strains after sub-MIC peptide exposure. Data presented as log₂ fold change relative to untreated control; mean ± SE of three biological replicates, with error bars indicating SE. **(C)** KEGG pathway enrichment analysis highlighting activation of stress response and protein folding pathways alongside repression of DNA replication and cell division pathways across the three *E. coli* strains. Data represent mean enrichment scores ± SD from three replicates, with error bars indicating SD; enrichment significance adjusted by Benjamini–Hochberg correction (FDR < 0.05).

To investigate the mode of bacterial killing, calcein leakage assays were conducted in LUVs preloaded with self-quenching levels of calcein dye (Fig. 6A). Peptide triggered extensive and rapid membrane permeabilization in bacterial-mimetic POPE/POPG vesicles, with dye leakage over 80% at 10 µM peptide concentration within 30 min. Dye leakage from mammalian-mimetic POPC/cholesterol vesicles was less than 20% under the same conditions, further establishing selective membrane disruption (Fig. 6A).

### Transcriptomic profiling reveals stress response and cellular disruption

RNA sequencing was done on three strains of *E. coli*—K-12 laboratory strain, uropathogenic (UPEC) strain, and multidrug-resistant (MDR) strain, after incubation with sub-MIC concentrations of peptide of each strain (1 μM, 2 μM, and 4 μM, respectively) for 1 h (Fig. 6B). Gene expression comparison revealed equal upregulation of stress response genes in all three strains. Specifically, the universal stress sigma factor rpoS (log₂ fold change ≈ 2.0, p = 0.008), and molecular chaperones dnaK (log₂ fold change ≈ 1.85, p = 0.012) and groEL (log₂ fold change ≈ 2.3, p = 0.005), favoring protein folding and damage fixation, had increased expression (Fig. 6B). Multidrug efflux pump proteins acrA (log₂ fold change ≈ 1.4, p = 0.015) and acrB (log₂ fold change ≈ 1.55, p = 0.01) were also induced, reflecting bacterial strategies for resisting peptide-induced stress (Fig. 6B). On the other hand, genes necessary for bacterial growth, such as dnaA (log₂ fold change ≈ –1.6, p = 0.02) and ftsZ (log₂ fold change ≈ –2.1, p = 0.007), had severe downregulation, indicating repression of cell division and DNA replication processes (Fig. 6B). These transcriptomic signatures were verified by KEGG pathway enrichment analysis, which indicated strong activation of stress response, protein folding, and efflux pump pathways with top pathway enrichment scores in the K-12 strain and slightly lower in the MDR strain, which is suggestive of strain-specific modulation (Fig. 6C). DNA replication and cell division pathways were downregulated uniformly in all the strains, but with slightly more downregulation observed in the lab and UPEC strains (Fig. 6C).

## Discussion

This manuscript outlines the rational design and full characterization of an optimized artificial AMP with high membrane selectivity, potent antibacterial activity, and minimal host cytotoxicity. Our approach involved the use of state-of-the-art biophysical techniques, microbiological tests, and transcriptomics to unravel a wide-ranging understanding of the structural dynamics of the peptide, its mechanism of action, and its therapeutic potential.

CD and SRCD measurements indicated that the peptide is mostly a random coil under conditions of water but becomes a stable α-helix when challenged by bacterial membrane-mimetic lipids (POPE/POPG). The conformational variability is characteristic of most natural AMPs and is essential in enabling membrane insertion and disruption. The thermal denaturation profile revealed, too, an unfolding cooperative transition at approximately 58°C, typical of stable helical structure in membrane environments, which must enhance peptide efficacy. SPR and ITC analyses revealed that the peptide binds to bacterial-like membranes with high affinity (nanomolar K_D_) and that binding to mammalian-like membranes (POPC/cholesterol) was considerably weaker (micromolar K_D_), demonstrating excellent selectivity. The positive binding thermodynamics with enthalpic and entropic contributions indicate a shared electrostatic attraction to negatively charged bacterial lipids and hydrophobic stabilization of membrane insertion.

Biologically, the peptide was highly active against typical, clinical, and drug-resistant strains of *E. coli* with MIC values of 2–8 µM. Importantly, it also exhibited potent activity against all six clinically significant ESKAPE pathogens, demonstrating broad-spectrum potential against major nosocomial threats. Quick lethality within two hours at fourfold MIC indicates its potential for clinical applications with rapid bacterial clearance. Interestingly, repeated sub-MIC exposure did not produce resistance by 10 consecutive passages, which represents a major advantage over conventional antibiotics and echoes the peptide’s action mechanism against the fundamental integrity of bacterial membranes, an action model difficult for bacteria to evade by mutations.

The peptide also exhibited a very good safety profile, showing little cytotoxicity in human cell lines at concentrations many-fold higher than those necessary for antimicrobial activity. Moderate membrane damage was noted at very high concentrations (50 µM), as would be predicted by the strong lipid selectivity noted biophysically. The peptide reduced the release of pro-inflammatory cytokines (TNF-α and IL-6) from THP-1 macrophages in a dose-dependent fashion. While these data suggest potential immunomodulatory activity, the reductions may arise either from direct interactions with immune signaling pathways or from indirect stress-related responses secondary to peptide treatment. This effect aligns with the well-established immunomodulatory functions of the parent peptide LL-37^35^, which has been shown to interact with immune receptors such as formyl peptide receptor-like 1 (FPRL1), P2X7, and TLRs (e.g., TLR4), leading to modulation of cytokine responses and macrophage polarization^36^. Other synthetic AMPs, including RiLK1 and cathelicidin-derived analogs^37^, have also been reported to attenuate pro-inflammatory cytokine production in infection models, highlighting that immunomodulation is a shared but multifactorial feature of AMPs. Notably, the immunomodulatory profile and selectivity of our peptide closely parallel those of LL-37, while showing enhanced antimicrobial potency and broader activity against resistant strains. While the exact receptor interaction of our peptide is not yet defined, the similarity in immunomodulatory effects supports the hypothesis that it may engage similar or overlapping signaling pathways. Further studies will be required to dissect whether the observed cytokine reduction results primarily from direct receptor engagement or from indirect stress-related mechanisms.

Mechanistic insight into the bactericidal action of the peptide was achieved through the use of membrane permeabilization assays. The peptide caused considerable dye leakage of calcein from bacterial-mimetic vesicles, establishing efficient membrane disruption, without damaging mammalian-like vesicles, consistent with selective killing. RNA sequencing of peptide-treated bacteria identified a multi-dimensional stress response through upregulation of defense chaperones (e.g., rpoS, dnaK, groEL) and efflux pump subunits (acrAB), indicating bacterial attempts to counteract membrane and proteotoxic stress. Concurrently, DNA replication and cell division genes were also decreased, suggesting a more widespread cellular disruption secondary to membrane disruption, for instance, loss of membrane potential or energy deprivation. These transcriptomic findings provide a deeper understanding of bacterial defense mechanisms and offer valuable targets for combination therapy with synergy or additional peptide optimization to achieve the highest efficacy and lowest risk of resistance.

Collectively, these findings highlight the power of a rational, mechanism-guided design strategy that optimizes amphipathicity, charge, and membrane selectivity to generate a potent yet safe AMP. The integration of biophysical profiling, functional assays, and whole-transcriptome analysis sets a new standard for AMP development by linking molecular mechanisms to cellular responses. The designed peptide’s broad-spectrum activity against *E. coli* and ESKAPE pathogens, low host cytotoxicity, LL-37–inspired immunomodulatory activity, and resistance-refractory mechanism underscore its promise as a next-generation antimicrobial candidate. Moreover, transcriptomic insights into bacterial stress responses reveal potential avenues for combination therapies and further peptide optimization to overcome adaptive resistance. Ultimately, this work establishes a comprehensive framework for AMP development and paves the way for future preclinical and clinical applications.

## Conclusion

This work describes an extensive evaluation of a rationally designed antimicrobial peptide as a promising candidate for further preclinical development. Its ability to fold from an aqueous disordered conformation into an ordered α-helical structure upon binding to bacterial membranes underlies its mechanism of selective targeting. Biophysical characterization confirmed its preferential binding to bacterial-mimicking lipids while showing minimal activity toward mammalian membrane models, thereby reducing host cell toxicity. Functionally, the peptide demonstrated potent and fast-acting bactericidal activity against both control and drug-resistant strains of *E. coli* as well as clinically relevant ESKAPE pathogens (*Enterococcus faecium, Staphylococcus aureus, Klebsiella pneumoniae, Acinetobacter baumannii, Pseudomonas aeruginosa,* and *Enterobacter* spp.). Compared with the parent peptide LL-37, our designed AMP consistently achieved 33–67% lower MICs and 2–3 fold faster bacterial killing kinetics across *E. coli* strains and a broad panel of Gram-negative and Gram-positive species, including ESKAPE pathogens. Under our experimental conditions, no resistance was observed over 10 passages. In addition, the peptide reduced pro-inflammatory cytokine production, suggesting potential immunomodulatory effects that may complement its antibacterial action. Transcriptomic profiling provided insights into bacterial stress responses and pathway changes following peptide treatment, offering valuable information for future optimization to maximize antimicrobial effectiveness and limit resistance development.

Together, these findings highlight the designed peptide as a promising lead for future translational studies targeting multidrug-resistant *E. coli* and ESKAPE pathogens with improved potency and rapid bactericidal activity compared to LL-37. As a next step, we plan to conduct comprehensive in vivo efficacy studies, apply advanced visualization techniques such as SEM, TEM, and DAPI/PI staining, perform detailed pharmacokinetic and safety assessments, and carry out systematic comparisons with benchmark antibiotics to further establish the therapeutic potential of this novel antimicrobial peptide.

## Supplementary Information

Below is the link to the electronic supplementary material.

Supplementary Material 1

## Data Availability

All data generated or analyzed during this study are included in this published article.
