# Supplementary material for: Biophysical and transcriptomic characterization of LL-37-derived antimicrobial peptide targeting multidrug-resistant Escherichia coli and ESKAPE pathogens
Source: Sci Rep. 2025 Oct 16;15:36126. doi: 10.1038/s41598-025-22890-7 (PMC12533254; doi:10.1038/s41598-025-22890-7)
Supplement: Supplementary file 1 — Supplementary Material 1 [file 41598_2025_22890_MOESM1_ESM.docx]

**Title:** *Biophysical and Transcriptomic Characterization of LL-37-Derived Antimicrobial Peptide Targeting Multidrug-Resistant Escherichia coli and ESKAPE Pathogens*

**Authors:**

Omar Eladl*
Faculty of Pharmacy, King Salman International University (KSIU), Ras Sudr, South Sinai, Egypt

*Corresponding author

omarsobhyeladl@gmail.com


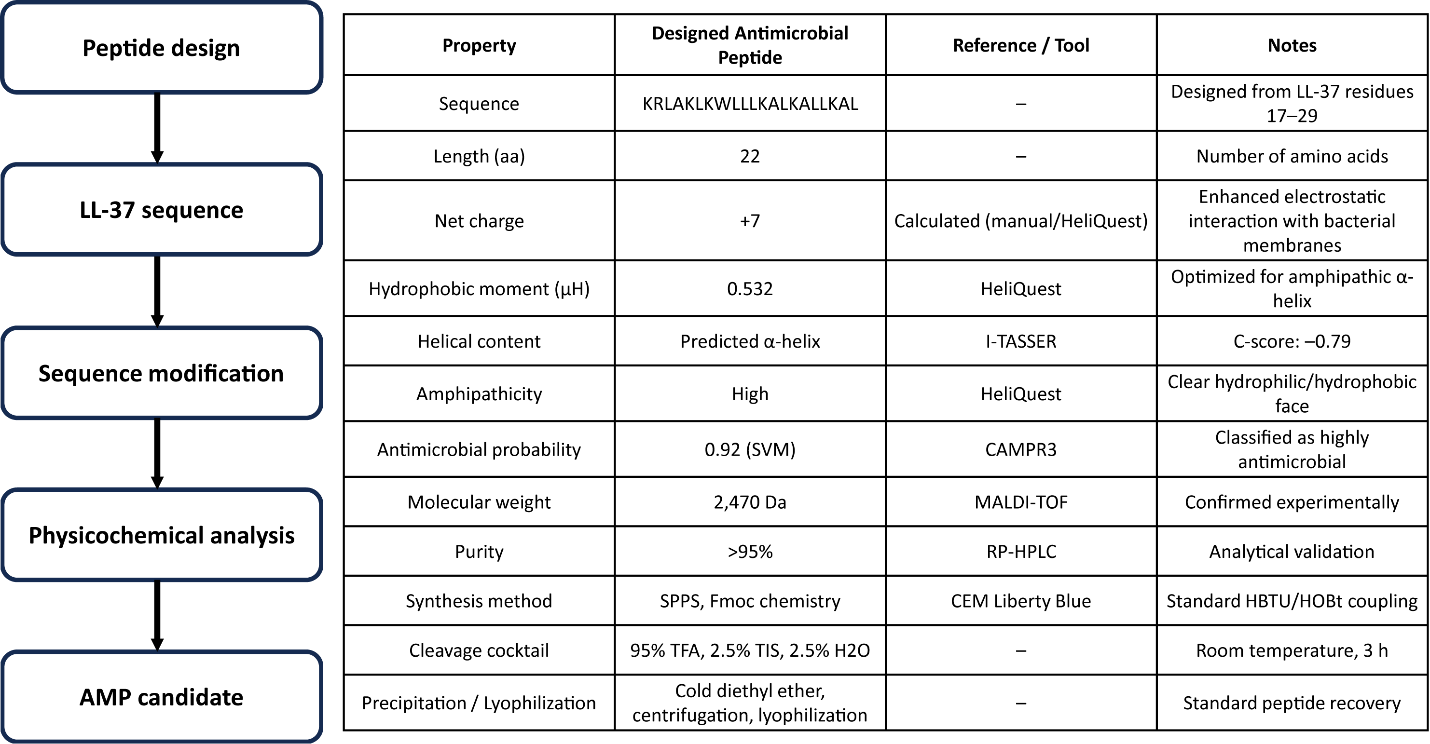


**Figure S1.** Design and characterization of the antimicrobial peptide.
Workflow of rational peptide design and summary of key physicochemical properties, predicted structure, antimicrobial potential, and synthesis details.


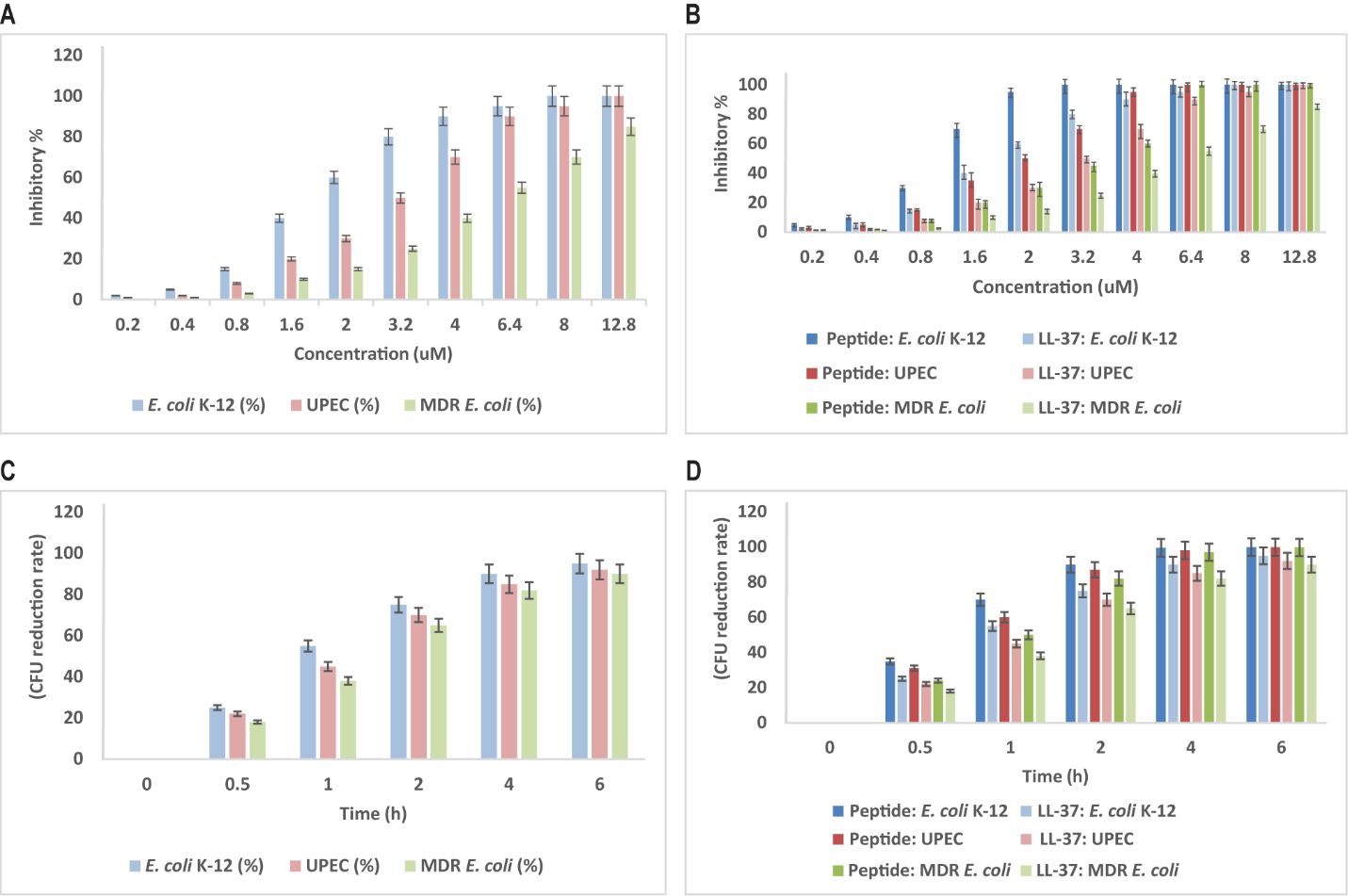


**Figure S2.** Comparative antimicrobial activity of the designed peptide and LL-37 against E. coli strains.
**(A)** Minimum inhibitory concentrations (MICs) of LL-37 against laboratory, uropathogenic, and multidrug-resistant E. coli. Data are expressed as mean ± SD from three independent experiments; error bars represent SD.
**(B)** Bar chart comparison of MICs between the designed peptide and LL-37 for the three E. coli strains. Error bars represent SD. The designed peptide consistently shows lower MIC values, indicating higher potency.
**(C)** Time-kill curves for LL-37 at four times the MIC against the three E. coli strains. Percent bacterial survival was determined at 0, 0.5, 1, 2, 4, and 6 hours. Error bars indicate SD.
**(D)** Time-kill curves for the designed peptide under identical conditions. Error bars indicate SD. The peptide demonstrates faster and more complete bacterial clearance compared to LL-37. Statistical significance: p < 0.001.


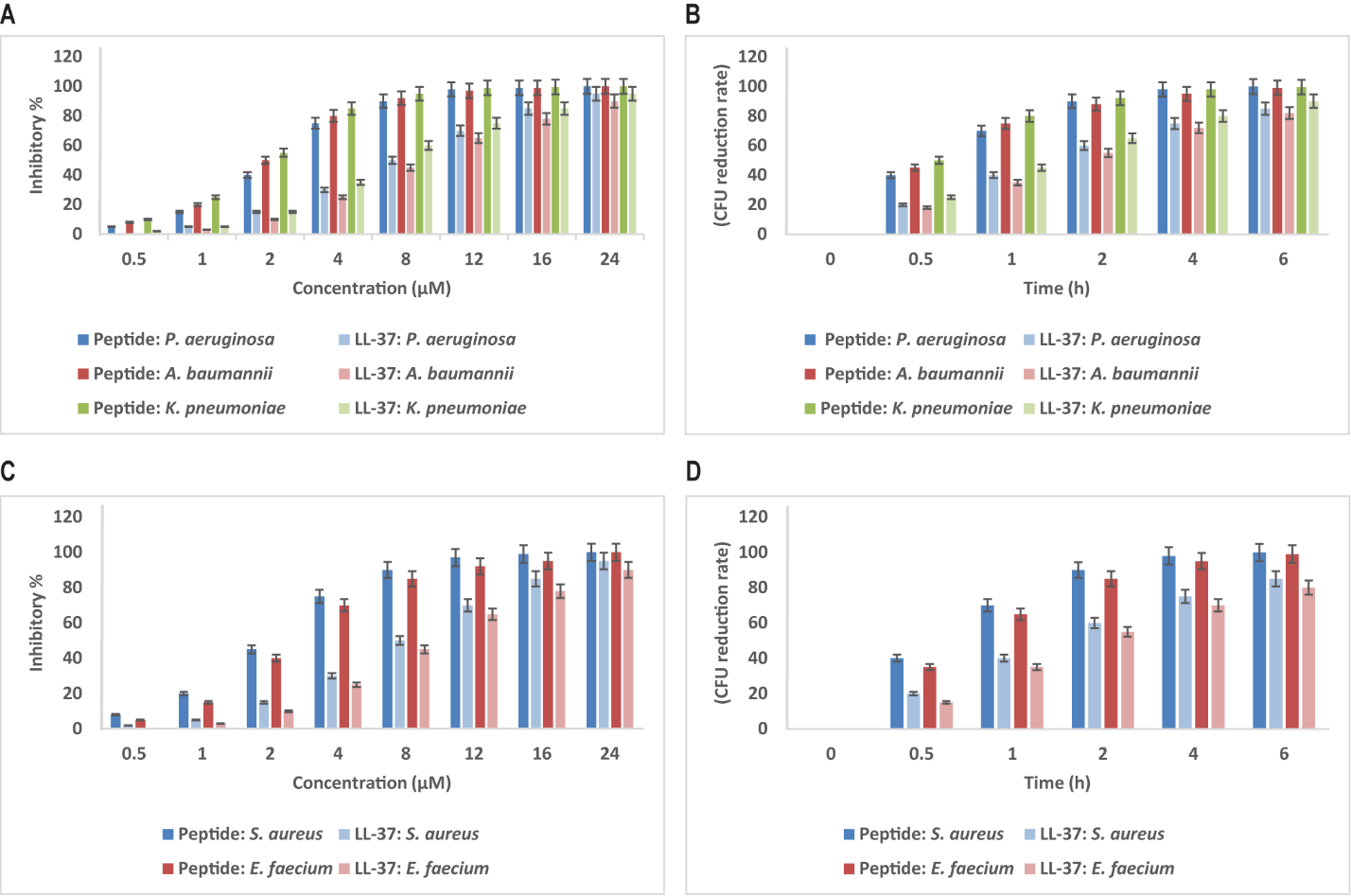


**Figure S3.** Antimicrobial activity of the designed peptide and LL-37 against Gram-positive and Gram-negative pathogens.
**(A)** MICs of the designed peptide and LL-37 against Gram-positive bacteria (Staphylococcus aureus and Enterococcus faecium). Data are mean ± SD from three independent experiments; error bars represent SD. The designed peptide displays lower MICs relative to LL-37.
**(B)** Time-kill assays at four times the MIC for the peptide and LL-37 against the Gram-positive bacteria. Percent bacterial survival was determined at 0, 0.5, 1, 2, 4, and 6 hours; error bars indicate SD. The designed peptide achieves faster and more complete killing compared to LL-37.
**(C)** MICs of the designed peptide and LL-37 against Gram-negative bacteria (Pseudomonas aeruginosa, Acinetobacter baumannii, and Klebsiella pneumoniae). Values are mean ± SD from three independent experiments; error bars represent SD. The designed peptide consistently shows lower MICs, indicating enhanced potency.
**(D)** Time-kill assays at four times the MIC for the peptide and LL-37 against the Gram-negative bacteria. Percent bacterial survival was determined at 0, 0.5, 1, 2, 4, and 6 hours; error bars indicate SD. The designed peptide demonstrates faster bactericidal kinetics and higher total killing relative to LL-37. Statistical significance: p < 0.001.
